# Supplementary material for: Protective Effects of Isostrictiniin Against High-Fat, High-Sugar Diet-Induced Steatosis in MASLD Mice via Regulation of the AMPK/SREBP-1c/ACC Pathway
Source: Nutrients. 2024 Nov 13;16(22):3876. doi: 10.3390/nu16223876 (PMC11597722; doi:10.3390/nu16223876)
Supplement: Supplementary file 1 [file nutrients-16-03876-s001.zip › nutrients-3292501-supplementary.pdf]

Supplementary Materials

This supplementary materials file contains the following contents:

Supplementary Tables	Table S1 to S8
Supplementary Figures	Figure S1 to S5

Table S1: Mice weight of mice at 0-4 weeks

Group	n	0week	1week	2week	3week	4week (g)
NC	8	25.66±1.44	29.79±1.86	34.10±2.03	37.10±2.08	40.56±1.43
HFSD	40	25.86±1.33	31.30±1.60	37.84±1.82	43.15±1.78	45.79±1.76

Data are presented as mean ± SDs.

Table S2: Mice weight of mice at 4-8 weeks

Group	4week	5week	6week	7week	8week (g)
NC	40.56±1.43	42.39±1.69	44.39±1.76	45.94±1.80	47.34±1.78
HFSD	45.73±1.48	49.10±1.93	53.08±1.39	56.13±1.60	59.04±1.66
ATC (10mg/kg)	45.15±1.58	47.16±1.94	49.43±2.29	51.79±2.12	54.04±2.01
ITN (25mg/kg)	46.43±1.09	49.58±1.36	52.21±1.73	54.45±1.78	57.31±1.42
ITN (50mg/kg)	45.96±2.35	48.73±1.77	51.50±1.72	53.99±1.28	56.65±1.40
ITN (100mg/kg)	45.68±2.18	48.68±1.08	50.88±1.31	52.79±1.21	55.06±1.30

Data are presented as mean ± SDs (n = 8).

Table S3: Effects of ITN on body weight, liver weight, liver index, serum AST and ALT in mice

Group	body weight /g	liver weight /g	liver index /%	AST/U·L^{-1}	ALT/U·L^{-1}
NC	47.34±1.78	1.56±0.10	3.30±0.18	113.21±10.73	34.04±1.75
HFSD	59.04±1.66$^{##}$	2.61±0.12$^{##}$	4.42±0.24$^{##}$	258.95±32.70$^{##}$	141.01±27.32$^{##}$
ATC (10mg/kg)	54.04±2.01**	1.86±0.09**	3.45±0.17**	133.39±27.88**	40.41±5.91**
ITN (25mg/kg)	57.31±1.42*	2.30±0.15**	4.01±0.23**	172.86±27.44**	80.11±27.28*
ITN (50mg/kg)	56.65±1.40**	2.00±0.05**	3.53±0.08**	157.21±32.55**	70.01±21.02*
ITN (100mg/kg)	55.06±1.30**	1.90±0.12**	3.45±0.28**	142.94±22.14**	56.34±10.95**

Data are presented as mean ± SDs (n = 8). $^{##}P < 0.01$ vs. NC; *$P < 0.05$, **$P < 0.01$ vs. HFSD.

Table S4: Effects of ITN on serum TG, TC, LDL-C and HDL-C in mice

Group	TG/mmol·L^{-1}	TC/mmol·L^{-1}	LDL-C/mmol·L^{-1}	HDL-C/mmol·L^{-1}
NC	1.13±0.28	2.60±0.28	0.18±0.04	2.84±0.12
HFSD	2.43±0.59$^{##}$	3.63±0.45$^{##}$	0.36±0.05$^{##}$	1.72±0.24$^{##}$
ATC (10mg/kg)	1.41±0.37**	2.77±0.53**	0.23±0.07**	2.79±0.31**
ITN (25mg/kg)	1.83±0.30**	3.02±0.62*	0.29±0.07**	2.56±0.46*
ITN (50mg/kg)	1.58±0.35**	2.98±0.41*	0.27±0.04**	2.67±0.22**
ITN (100mg/kg)	1.42±0.39**	2.89±0.65**	0.25±0.06**	2.77±0.42**

Data are presented as mean ± SDs (n = 8). $^{##}P < 0.01$ vs. NC; $^{*}P < 0.05$, $^{**}P < 0.01$ vs. HFSD.

Table S5: Effects of ITN on FBG, FINS and HOMA-IR in mice

Group	FBG/ mmol·L^{-1}	FINS/mIU·L^{-1}	HOMA-IR
NC	3.75±0.57	3.15±0.49	0.52±0.10
HFSD	8.08±0.72$^{##}$	4.10±0.48$^{##}$	1.46±0.16$^{##}$
ATC (10mg/kg)	4.28±0.92**	3.31±0.69**	0.62±0.17**
ITN (25mg/kg)	7.20±0.52*	3.76±0.70	1.21±0.28*
ITN (50mg/kg)	6.61±0.50**	3.55±0.50	1.04±0.17**
ITN (100mg/kg)	5.64±0.93**	3.41±0.95*	0.84±0.21**

Data are presented as mean ± SDs (n = 8). $^{##}P < 0.01$ vs. NC; $^{*}P < 0.05$, $^{**}P < 0.01$ vs. HFSD.

Table S6: Effects of ITN on IL-6, TNF-α and IL-1β in liver tissue of mice

Group	TNF-α/ng·mL^{-1}	IL-6/ng·mL^{-1}	IL-1β/ng·mL^{-1}
NC	1.54±0.58	10.70±2.67	1.79±0.48
HFSD	3.44±0.80$^{##}$	18.10±1.87$^{##}$	3.69±1.24$^{##}$
ATC (10mg/kg)	1.70±0.77**	11.76±3.17**	1.91±0.47**
ITN (25mg/kg)	2.44±0.59**	13.86±1.63**	2.33±0.45*
ITN (50mg/kg)	2.21±0.54**	12.98±1.31**	2.16±0.48**
ITN (100mg/kg)	2.04±0.65**	12.55±2.21**	2.03±0.29**

Data are presented as mean ± SDs (n = 8). $^{##}P < 0.01$ vs. NC; $^{*}P < 0.05$, $^{**}P < 0.01$ vs. HFSD.

Table S7: Effects of ITN on SOD, MDA and GSH-PX in liver tissue of mice

Group	SOD/U·mgprot^{-1}	MDA/μmmol·gprot^{-1}	GSH-Px/U·mgprot^{-1}
NC	469.23±46.25	4.06±0.62	1006.84±153.38
HFSD	345.52±60.33$^{##}$	9.55±2.45$^{##}$	615.72±118.37$^{##}$
ATC (10mg/kg)	446.50±45.89**	5.21±1.01**	929.11±134.44**
ITN (25mg/kg)	364.02±52.33	6.35±0.95	703.24±160.00
ITN (50mg/kg)	426.49±43.63*	5.61±1.11**	792.19±172.17*
ITN (100mg/kg)	440.51±70.61**	5.46±0.81**	873.76±249.20**

Data are presented as mean ± SDs (n = 8). $^{##}P < 0.01$ vs. NC; $^{*}P < 0.05$, $^{**}P < 0.01$ vs. HFSD.

Table S8: Effects of ITN on proteins expression of p-AMPK/AMPK、SREBP-1c、ACC in the liver tissues of mice

Group	p-AMPK/AMPK	SREBP-1c/GAPDH	ACC/GAPDH
NC	1.10±0.07	0.65±0.05	0.57±0.09
HFSD	0.54±0.02##	1.09±0.17##	0.94±0.09##
ATC (10mg/kg)	1.04±0.05**	0.71±0.11**	0.60±0.05**
ITN (25mg/kg)	0.71±0.03*	0.89±0.08*	0.80±0.03*
ITN (50mg/kg)	0.85±0.13**	0.85±0.11*	0.74±0.11**
ITN (100mg/kg)	0.98±0.11**	0.75±0.08**	0.64±0.08**

Data are presented as mean ± SDs (n = 3). ##*P* < 0.01 vs. NC; **P* < 0.05, ***P* < 0.01 vs. HFSD.

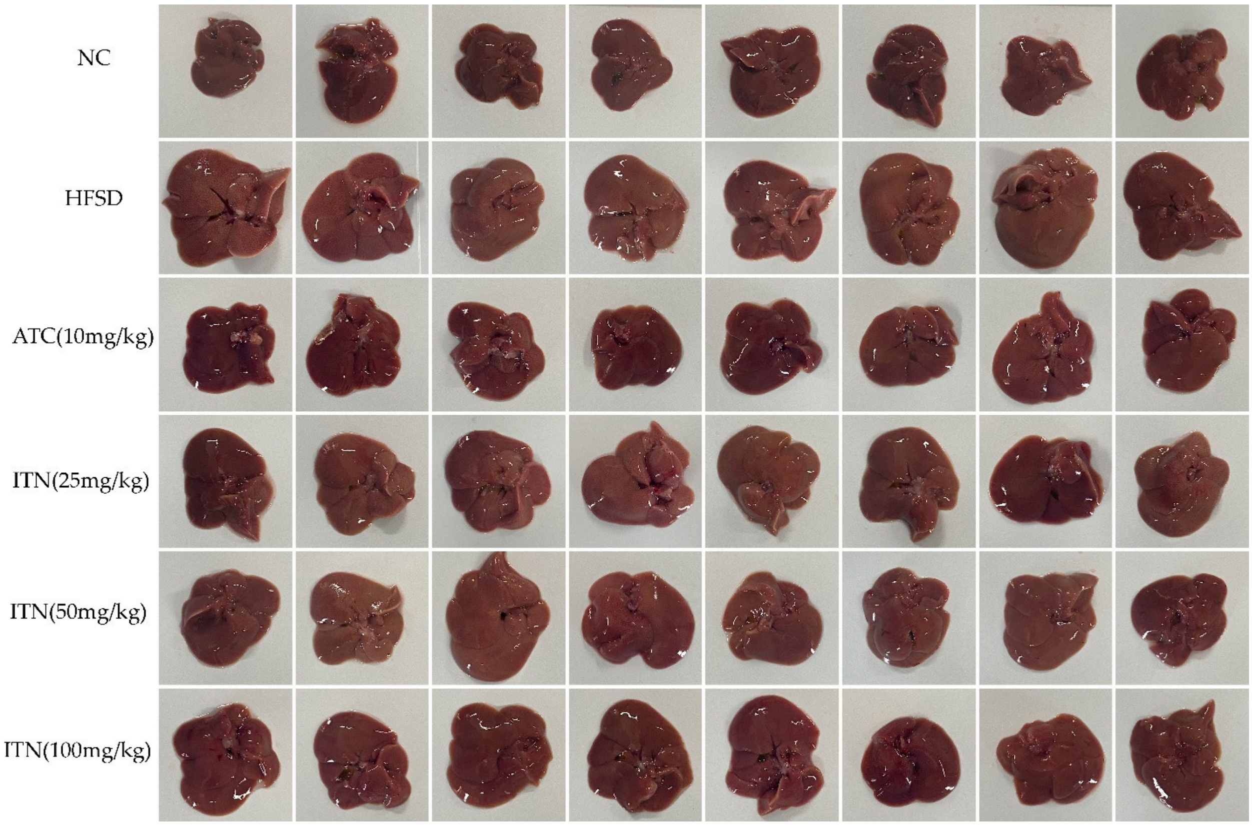

Figure S1. The gross liver tissues from the groups of mice at the end of the experiment

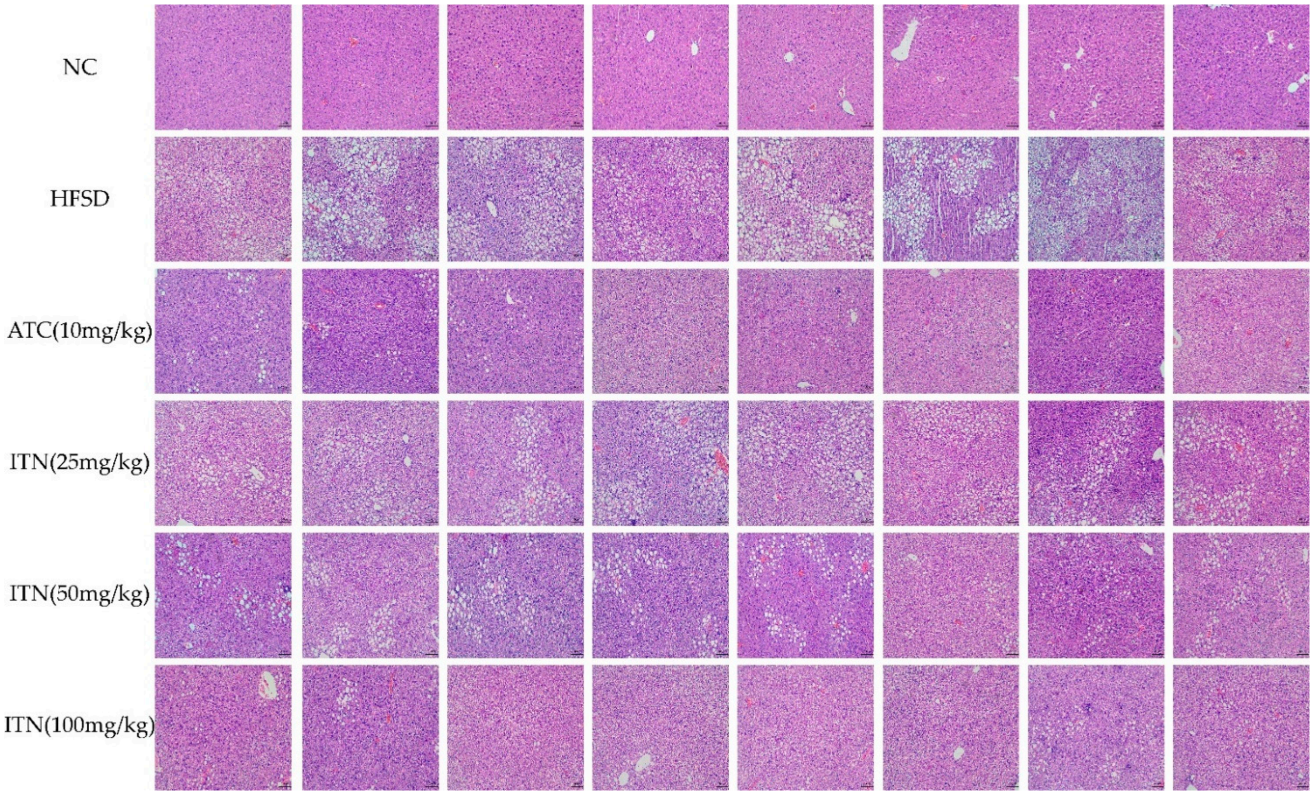

Figure S2.H&E staining of mouse liver sections

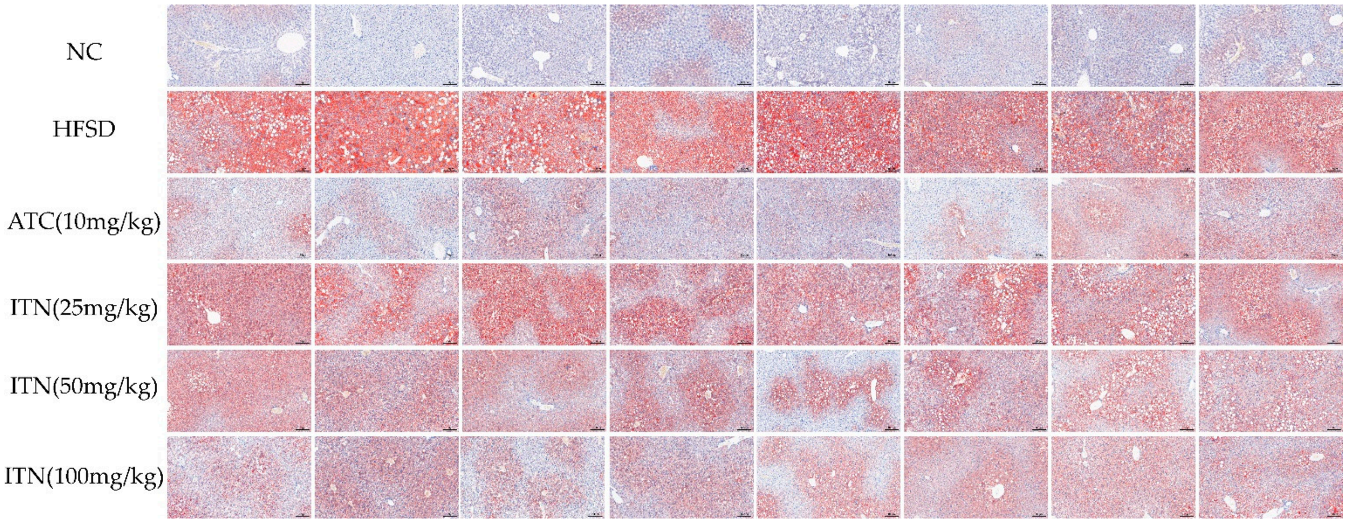

Figure S3. Oil red O staining of mouse liver sections

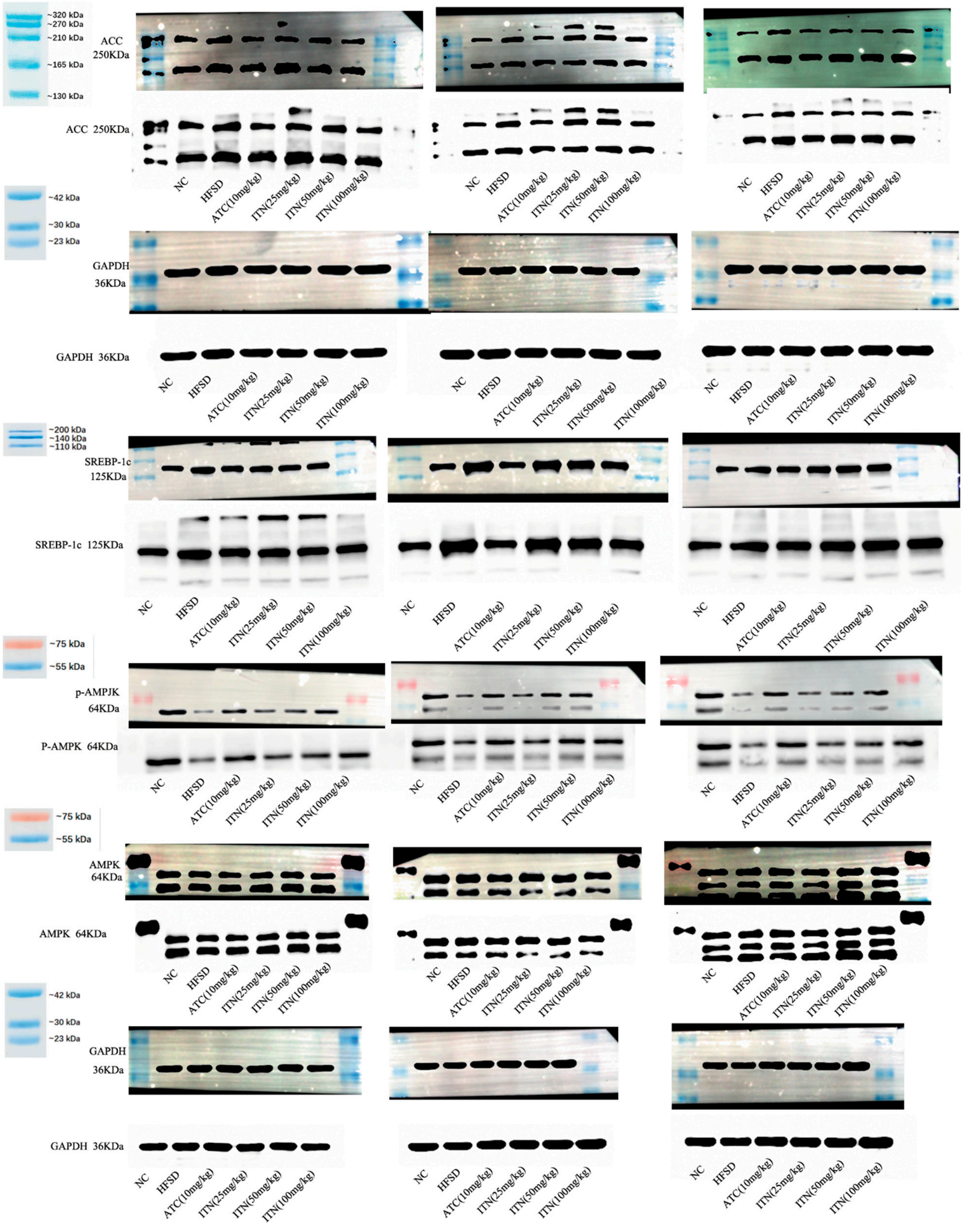

Figure S4. Effects of ITN on proteins expression of p-AMPK/AMPK, SREBP-1c and ACC in the liver tissues of mice

The HPLC conditions were as follows： Phenomenex Gemini-NX C18 column (250 mm×4.6 mm, 5μm), the mobile phase of A(acetonitrile) and B (0.2%phosphoric acid, v/v) with a gradient elution (0~35min, 5%-15%A; 35~65 min, 15%-18%A; 65~70min, 18%-20%A; 70~75 min, 20%-5%A), flow rate at 1.0 mL/min, column temperature at 30℃, and detection wavelength at 266 nm. Parallel tests were performed three times. The purity of ITN was 95.94%, 95.97% and 95.99% by HPLC normalization method. The chromatogram is as follows:

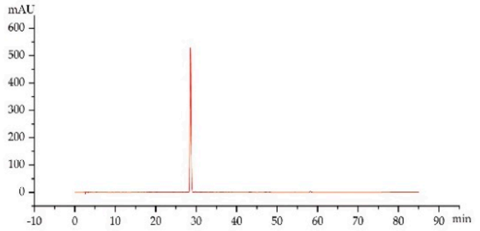

Serial number	minutes	Area of peak	Height of peak	Peak width	Factor of symmetry	Area of peak %	Type
1	2.665	40.3	7.8	0.0843	0.758	0.414	BV
2	2.778	86.9	6.5	0.1751	0.237	0.892	VV
3	3.268	51	3.5	0.1818	8.976	0.524	VB
4	3.406	33.7	2.4	0.2053	0.362	0.346	BB
5	28.789	9344.8	522.5	0.2768	0.776	95.939	BB
6	29.968	40.7	2.1	0.2875	0.953	0.418	BB
7	47.32	44.3	2	0.3263	0.833	0.455	BB
8	58.4	98.5	3.6	0.4177	0.857	1.012	BB

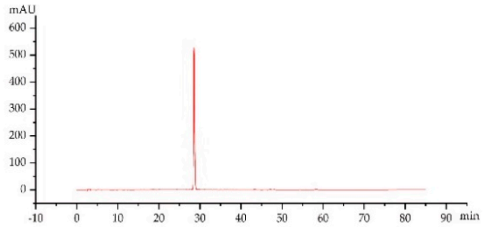

Serial number	minutes	Area of peak	Height of peak	Peak width	Factor of symmetry	Area of peak %	Type
1	2.666	38.8	7.7	0.0809	0.744	0.399	BV
2	2.777	86.8	6.4	0.1751	0.24	0.892	VV
3	3.268	50.7	3.5	0.1773	8.733	0.521	VB
4	3.406	32.2	2.4	0.1962	0.378	0.33	BB
5	28.783	9343.4	520.2	0.2795	0.774	95.968	BB
6	29.958	40.5	2.2	0.2891	0.938	0.416	BB
7	47.204	44.2	2.1	0.3236	0.849	0.454	BB
8	58.186	99.2	3.6	0.4152	0.816	1.019	BB

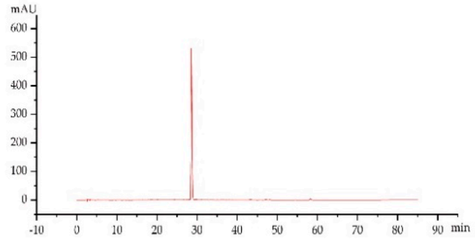

Serial number	minutes	Area of peak	Height of peak	Peak width	Factor of symmetry	Area of peak %	Type
1	2.663	39.4	7.8	0.0811	0.728	0.405	BV
2	2.776	86.4	6.5	0.1742	0.243	0.888	VV
3	3.264	50.3	3.5	0.1758	8.498	0.516	VB
4	3.394	29.6	2.4	0.1821	0.345	0.304	BB
5	28.575	9343.1	529	0.2741	0.777	95.988	BB
6	29.721	40.5	2.2	0.2828	0.926	0.416	BB
7	47.146	45.1	2	0.3308	0.851	0.463	BB
8	58.234	99.2	3.6	0.4123	0.816	1.019	BB

Figure S5. HPLC graph of INT
